# Effect of the upward curvature of toe springs on walking biomechanics in humans

**DOI:** 10.1038/s41598-020-71247-9

**Published:** 2020-09-17

**Authors:** Freddy Sichting, Nicholas B. Holowka, Oliver B. Hansen, Daniel E. Lieberman

**Affiliations:** 1 Department of Human Locomotion, Chemnitz University of Technology, Chemnitz, Germany; 2 Department of Anthropology, University At Buffalo, Buffalo, NY USA; 3 Department of Human Evolutionary Biology, Harvard University, Cambridge, MA USA

**Keywords:** Musculoskeletal system, Ligaments, Biomechanics

## Abstract

Although most features of modern footwear have been intensively studied, there has been almost no research on the effects of toe springs. This nearly ubiquitous upward curvature of the sole at the front of the shoe elevates the toe box dorsally above the ground and thereby holds the toes in a constantly dorsiflexed position. While it is generally recognized that toe springs facilitate the forefoot’s ability to roll forward at the end of stance, toe springs may also have some effect on natural foot function. This study investigated the effects of toe springs on foot biomechanics in a controlled experiment in which participants walked in specially-designed sandals with varying curvature in the toe region to simulate toe springs ranging from 10 to 40 degrees of curvature. Using inverse dynamics techniques, we found that toe springs alter the joint moments and work at the toes such that greater degrees of toe spring curvature resulted in lower work requirements during walking. Our results help explain why toe springs have been a pervasive feature in shoes for centuries but also suggest that toe springs may contribute to weakening of the foot muscles and possibly to increased susceptibility to common pathological conditions such as plantar fasciitis.

## Introduction

**Figure 1:**
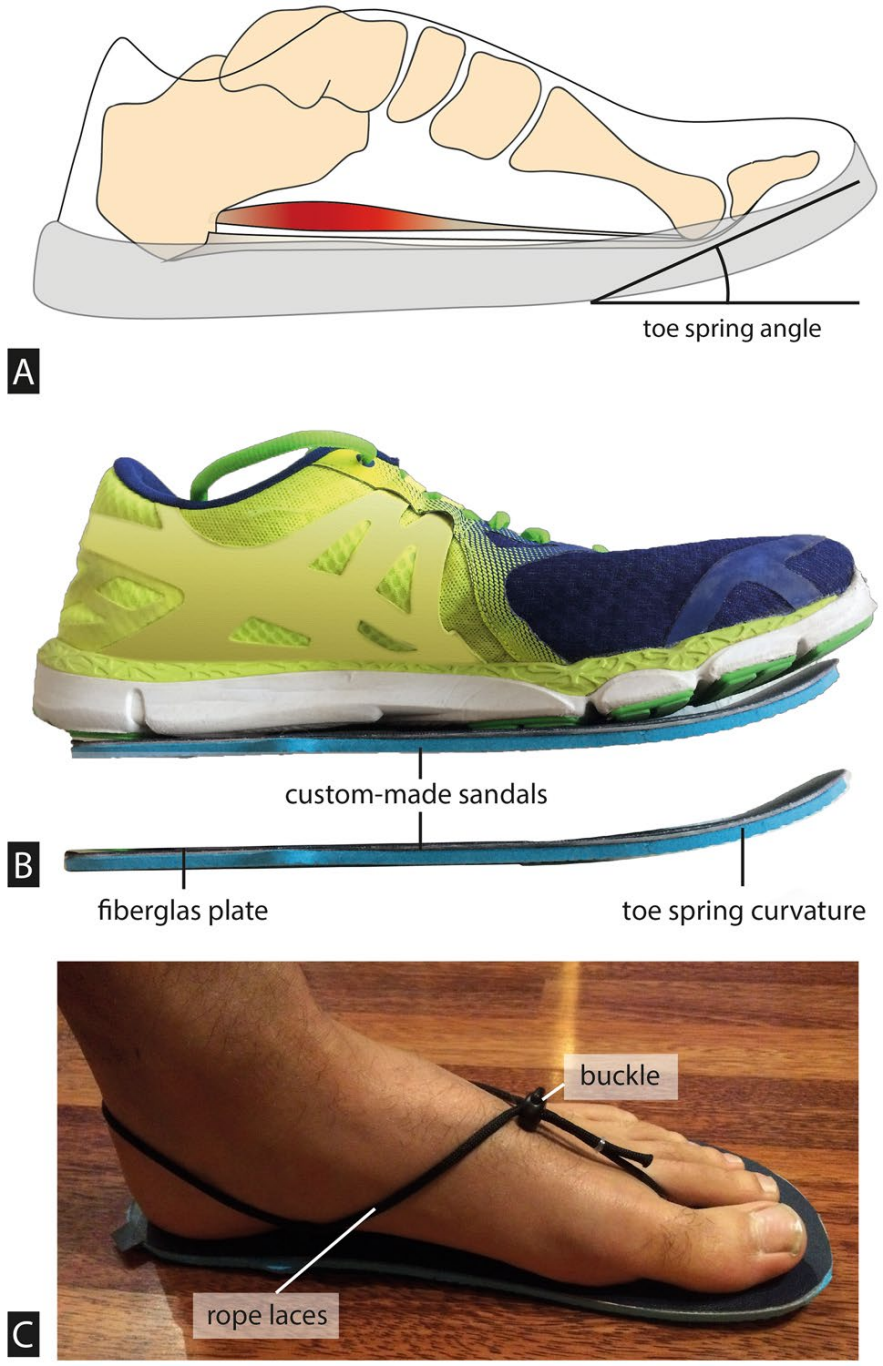
A toe spring describes the curve upward of the sole of a shoe. (**A**) The upward curvature below the metatarsal heads orients the toe box dorsally relative to the rest of the shoe. (**B**) Custom-made sandals with varying degrees of toe spring angle were manufactured to mimic the stiffness and shape of toe springs commonly found in commercially available shoes. (**C**) The sandals were secured with minimal rope laces that could be adjusted by a buckle and did not restrict the placement of reflective markers.

Most humans today use footwear with numerous features that protect the sole of the foot and increase comfort. While many features have been intensively studied^1^, one nearly ubiquitous feature that has been almost entirely unstudied is the toe spring. This upward curvature of the sole of the shoe below the metatarsal heads orients the toe box dorsally relative to the rest of the shoe (Fig. 1). The toe spring is generally thought to help the forefoot roll forward during the propulsive phase of walking, between when the heel and the toes leave the ground. The benefits in terms of mechanical work of this rolling motion have already been demonstrated in footwear with curved, rocker-bottom surfaces^2–4^. Specifically, this rolling motion appears to reduce center of mass work, although the extent to which the conditions in these studies correspond to the toe springs in conventional shoes is unclear. To date, no experimental study has examined how the toe spring affects the way the human foot functions during gait, and how it may affect the foot’s vulnerability to injury.

**Figure 2:**
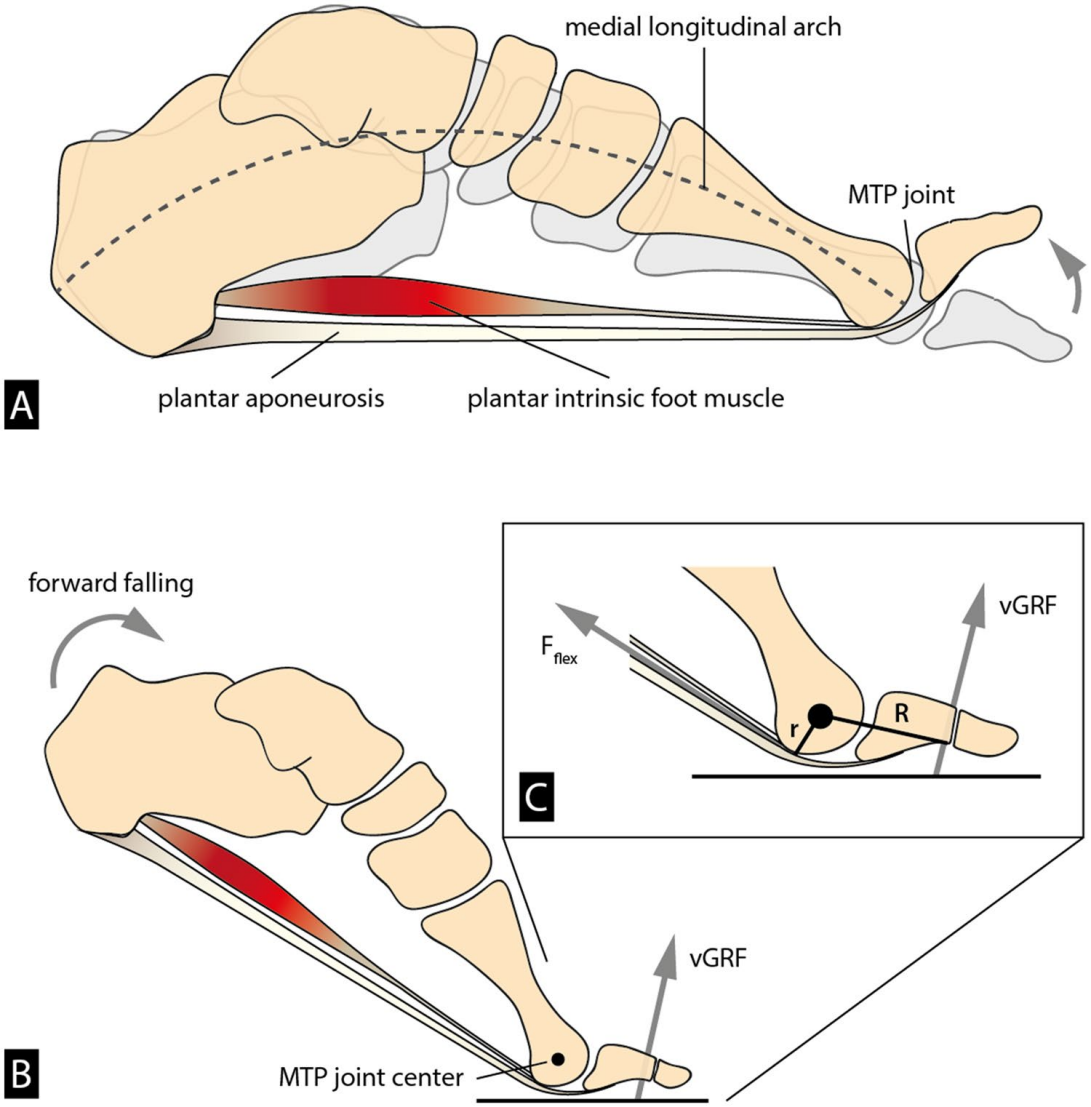
The ability to dorsiflex the toes relative to the rest of the foot at the metatarsophalangeal (MTP) joints during the propulsive phase is one of the key evolved features that enable humans to walk and run bipedally effectively and efficiently. (**A)** Dorsiflexion at the MTP joints helps stiffen the foot through a windlass mechanism. During this action, dorsiflexion of the toes creates tension in the plantar aponeurosis that tends to pull the calcaneus towards the metatarsal heads. This motion creates an upward force in the longitudinal arch. (**B**) During propulsive phase, the metatarsal heads and the distal phalanges are the only points of contact with the ground on the trailing leg and hence become load-bearing. As a result, the ground reaction force (vGRF) acts on the distal phalanges at a distance R from the MTP joint center to generate a moment that causes the MTP joints to dorsiflex. (**C**) The intrinsic flexor muscles are active (F_flex_) at the end of stance phase, balancing the dorsiflexion moments at the MTP joints (with r as the lever arm of the acting flexor muscles).

It is well established that the ability to dorsiflex the toes relative to the rest of the foot at the metatarsophalangeal (MTP) joints is one of the key evolved features that enable humans to walk and run bipedally effectively and efficiently. In addition to having shorter, straighter phalanges, human metatarsal heads are characterized by more dorsally oriented and mediolaterally broad articular surfaces compared to those of our closest relatives, the African apes^5^. During the propulsive phase of walking, the dorsally oriented metatarsal heads in the human forefoot are thought to increase the range of dorsiflexion motion at the MTP joints by providing more dorsal articular surface area on which the proximal phalangeal base can slide^6–10^. Although recent research shows that transverse splaying of the metatarsal heads helps stiffen the midfoot via the transverse tarsal arch^11^, it has long been argued that dorsiflexion at the MTP joints also helps stiffen the foot through a windlass mechanism^12^. During this action, dorsiflexion of the toes tightens the plantar aponeurosis, a broad sheet of highly fibrous tissue whose collagen fibers span the plantar aspect of the foot from the heel to the toes (for review see^13^). The increased tension on the plantar aponeurosis pulls the calcaneus and metatarsal heads towards each other, creating an upward force that elevates the longitudinal arch, counters compressive forces from above, and stiffens the foot as a whole (Fig. 2A). Recent research, however, challenges this traditional perspective of the windlass mechanism. In a static in vivo loading experiment, Welte et al.^14^ found that raising the longitudinal arch by dorsiflexing the toes actually decreases the longitudinal arch’s stiffness. In another static in vivo experiment, Farris et al.^15^ found that the windlass mechanism has little effect on longitudinal arch motion while the arch is experiencing the high loads associated with push-off. While these findings are compelling, further verification from dynamic in vivo locomotion is necessary, and the windlass mechanism remains a widely utilized model for understanding the functional significance of the longitudinal arch (e.g.,^16–18^).

Regardless of the extent to which the windlass is a passive stabilizing mechanism, a growing body of research has shown that the intrinsic foot muscles also play important roles in supporting the longitudinal arch and stabilizing the MTP joints^19–21^. During propulsive phase, the metatarsal heads and the distal phalanges are the only points of contact with the ground on the trailing leg and hence become load-bearing. As a result, the ground reaction force loads applied to the distal phalanges generate a moment that causes the MTP joints to dorsiflex (Fig. 2B). Electromyographic studies indicate that the intrinsic muscles of the foot, especially the flexor digitorum brevis and abductor hallucis, are active at the end of stance phase, balancing the dorsiflexion moments at the MTP joints (Fig. 2C)^19,22^. According to these findings, proper intrinsic foot muscle activity, therefore, acts in concert with passive mechanisms such as the windlass to maintain foot stability during propulsion.

Because of the role that intrinsic foot muscles play in stabilizing the forefoot, weakness or dysfunction of these muscles may be associated with a variety of overuse injuries including plantar fasciitis^23,24^. This pathological inflammation causes pain and immobility in more than 2 million patients each year in the United States, making it the most common condition encountered by podiatrists^25^. Etiologically, plantar fasciitis is recognized as an injury caused by excessive and repetitive loading of the foot’s longitudinal arch^26^. Recent evidence suggests that plantar fasciitis could be related to weak foot muscles that are not strong enough to provide foot stability, thus increasing strain in the plantar fascia, which wraps around the MTP joints, presumably affecting their stability^27^. Several lines of evidence suggest that weak foot muscles may be partly a consequence of features in modern shoes that support the longitudinal arch and passively stiffen the foot^21,28,29^. As these studies showed, individuals who habitually wear minimal footwear have intrinsic foot muscles with large cross sectional areas and dynamically stiffer longitudinal arches than individuals who habitually wear modern shoes. Weak intrinsic foot muscles may thus be an evolutionary mismatch caused by the foot not being entirely adapted for modern shoes^30^. Until recently, humans were either barefoot or wore minimal shoes. Although the first evidence for minimal footwear dates back to 10,000 years ago^31,32^, most shoes until very recently were minimal and did not have arch supports, cushioning, and other supportive features that increase comfort and reduce the work that the foot muscles have to do^33^.

Here we focus on how toe springs affect the foot’s ability to function as a stiff lever, especially during the propulsive phase of stance. While it is generally recognized that toe springs facilitate the forefoot’s ability to roll forward at the end of stance, toe springs may also have some effect on arch stiffness via the windlass mechanism. It is reasonable to hypothesize that toe springs continually engage the windlass mechanism by permanently orienting the toes in a dorsiflexed position when they might otherwise be in a neutral, horizontal position and thereby elevate the arch. Without a toe spring, loading the arch should cause a ‘reverse windlass’ effect in which the toes are plantarflexed as the arch is compressed during walking or running^12^. However, a toe spring could prevent that motion from occurring, effectively stiffening the arch by preventing compression. This stiffening effect should be pronounced at midstance, when the foot is loaded by body mass prior to dorsiflexion of the toes at heel lift. Following this traditional perspective of the windlass mechanism, a toe spring could thus passively reduce the need for intrinsic foot muscles to actively resist arch deformation. Another related effect that toe springs could have on the foot concerns energy loss at the MTP joints during the propulsive phase of each step. It is well established that the digital flexor muscles do a significant amount of work as the MTP joints dorsiflex during this phase^34^, and previous studies have estimated that the work done by the digital flexor muscles is proportional to the amount of MTP joint rotation during push-off^35^. By passively dorsiflexing the toes before push-off, a toe spring could thus decrease the total angle through which the toes rotate while these muscles are active. These effects on foot biomechanics would reduce the total work required of the intrinsic foot muscles, possibly helping to explain their observed atrophy in individuals who habitually wear modern shoes.

Although toe springs affect foot biomechanics during walking and running, this study explores how the toe spring affects intrinsic foot biomechanics during walking because it is the most common gait. While toe springs may have general effects on overall gait, as has been demonstrated in studies of prosthetic toe shape and shoe midsole stiffness^2,18^, here we focus on the immediate effect of toe springs on intrinsic foot biomechanics to test discrete hypotheses about how they potentially affect foot function. We focus on the medial longitudinal arch and the MTP joints during midstance and propulsive phase and use kinematic and force data to test the general hypothesis that shoes with a toe spring will affect stiffness of the foot-shoe-complex and the total work done at the MTP joints. We also test two specific hypotheses. Hypothesis 1 is that during midstance, the stiffness of the medial longitudinal arch will increase with greater toe spring angles since the dorsiflexed position of the toes activates the windlass mechanism. Hypothesis 2 is that during the propulsive phase, increasing toe spring angles will gradually decrease the total angle through which the toes rotate and subsequently decrease the total work at the MTP joint.

## Methods

### Participants

Data were collected from 13 participants (9 male, 4 female), ranging in age from 19 to 33 years old (mean ± SD: 22 ± 3.1 years). Average weight was 74 ± 7.5 kg and average height was 182 ± 6 cm. All participants were apparently healthy and had no current injuries or conditions that would cause gait abnormalities. Written informed consent was obtained from each subject. The study protocol was approved by Harvard’s Committee on the Use of Human Subjects and conducted in accordance with the Declaration of Helsinki.

### Footwear design

Participants walked on the treadmill barefoot and in four pairs of custom-made sandals with varying degrees of toe spring angle. The sandals consisted of a top sole, rubber outsole, foam midsole (thickness 2 mm), and curved fiberglass plate that ran the length of the sandal and curved upwards at the ball of the foot to the tip of the sandal (Fig. 1B). The upwards curvature under the toes was either 10°, 20°, 30° or 40°. The 10° condition was chosen as the lowest profile to ensure a minimum of natural foot roll-over during the propulsive phase. The sandals were secured with minimal rope laces that could be adjusted by a buckle and did not restrict the placement of reflective markers. Two sandal sizes were used, depending on the participant’s foot size (24 cm and 28 cm length). We chose to use sandals rather than shoes for this study because of their relative ease of construction, and because they allowed us to place a detailed marker set on the foot (see below).

The sandals were designed to mimic the stiffness and shape of toe springs commonly found in commercially available shoes^36^. Before the experiment, the bending stiffness of the sandal was measured with a uniaxial tensile and compression testing machine (Model HC 10, Zwick GmbH & Co. KG, Ulm, Germany). The test set-up for measuring shoe bending stiffness has been described in detail elsewhere^37^. In brief, the rearfoot portion of the sandal was clamped down on a fixed platform set to align the rotational axis of the machine with the anatomical MTP joint bending axis. The distance between the midpoint of the metatarsal axis and the force application line was 50 mm. The sandal was bent by lifting and lowering a shaft by 40 mm. Using the corresponding force to the deformation curve enabled the calculation of torque and the bending angle. The average bending stiffness was calculated based on the torque–angle curve from 10 loading cycles at 2 Hz. The measured average bending stiffness of the sandal was 6.38 ± 1.58 Nm/rad, being similar to the bending stiffness of modern shoes (Adidas adizero: 7.00 Nm/rad, Nike Zoom Streak 6: 9.4 Nm/rad)^38^.

### Experimental treatment

Participants walked on a split belt treadmill (Bertec Corporation, Columbus, OH, USA) instrumented with separate force plates under each belt, which were used to measure the ground reaction forces (GRF) in each leg individually for each step. The order of the sandals and barefoot condition were randomized. Before the barefoot walking trial, participants were also recorded standing still for 10 s. This was done to provide a neutral representation of each participant’s foot so that we could normalize the joint angles calculated during walking to each participant’s neutral foot posture, where joint angles were set at 0 degrees. Participants walked in each condition for a few minutes until they felt comfortable. They walked at a speed proportional to leg length as determined by a convention known as a Froude number that follows the principle of dynamic similarity^39^. A Froude number of 0.15 was chosen for each subject because it is a comfortable, moderate walking pace. Leg length was measured as the distance from a subject’s greater trochanter to the ground. Kinetic and kinematic data were collected simultaneously across a 30 s data collection period for each walking trial (barefoot, 10°, 20°, 30° and 40° sandal). Ten steps were exported for further analysis.

### Acquisition of kinematic and kinetic data

Motion data were captured at 200 Hz using an eight-camera 3D optoelectronic motion capture system (Oqus, Qualysis, Gothenburg, Sweden). GRF data were synchronously captured with the motion data at 2000 Hz using the Qualisys Track Management software (Qualisys, Gothenburg, Sweden). In order to quantify three-dimensional motions of the foot and shank, fifteen retro-reflective markers (12.0 mm diameter) were placed on each subject’s right knee, ankle, and foot. These were placed on bony landmarks defined by Leardini et al.^40^, which define the leg and foot as five separate segments: the shank (lower leg between the knees and ankle) the hallux (toes), metatarsals (forefoot), midfoot, and calcaneus (rearfoot).

### Data analysis

Marker trajectories and GRF data were exported to Visual3D (C-motion Inc., Germantown, MD, USA) for post-processing and analysis. A recursive fourth-order Butterworth low-pass filter (10 Hz cutoff frequency) was used to process both kinematic and GRF data. The same cutoff frequency was used for both GRF and kinematic data to avoid artifacts in inverse dynamics calculations that occur when different filter cutoff frequencies are used^41^. Contact time (from heel contact to toe-off) was calculated with a 50 N vertical GRF threshold. All data curves were time normalized to the stance phase duration for plotting and visual inspection.

### Joint kinematics

The geometrical definitions of MLA and MTP joint angle were based on skin-markers located on the calcaneus (Cal), sustentaculum tali (ST), base of the first metatarsal bone (FMB), head of the first metatarsal bone (FMH), and distal end of the first proximal phalanx (PM)^40,42^. The MLA and MTP joint measurements were calculated as angles between the projections of two vectors on the sagittal plane of the foot overall and the forefoot segment, respectively, as defined in Leardini et al.^40^. For the MLA angle, the vector on the proximal segment is bounded by marker Cal_proj and ST, where Cal_proj is the projection of Cal on the x–y plane of the foot. The vector on the distal segment is bounded by markers ST and FMH^42^. For the MTP joint, the vector on the proximal segment is bounded by marker FMB and FMH, and the vector on the distal segment is bounded by markers FMH and PM. To evaluate the effect of toe springs on arch kinematics, MLA and MTP joint angles were analyzed at 50% of stance phase, although peak forces acting on the arch occur later in stance during walking. This allowed us to isolate the toe spring effect on MLA deformation, since this is prior to the initiation of normal MTP dorsiflexion due to heel lift during walking. To evaluate the effect of toe springs on MTP joint kinematics during the propulsive phase, peak MTP joint dorsiflexion angle and the corresponding MLA angle, as well as peak MTP joint angular velocity, were quantified. Further, total range of MTP joint dorsiflexion angle was defined as the range through which the toes rotate from the moment when the COP aligns with the MTP joint to peak dorsiflexion angle.

### Joint kinetics

**Figure 3:**
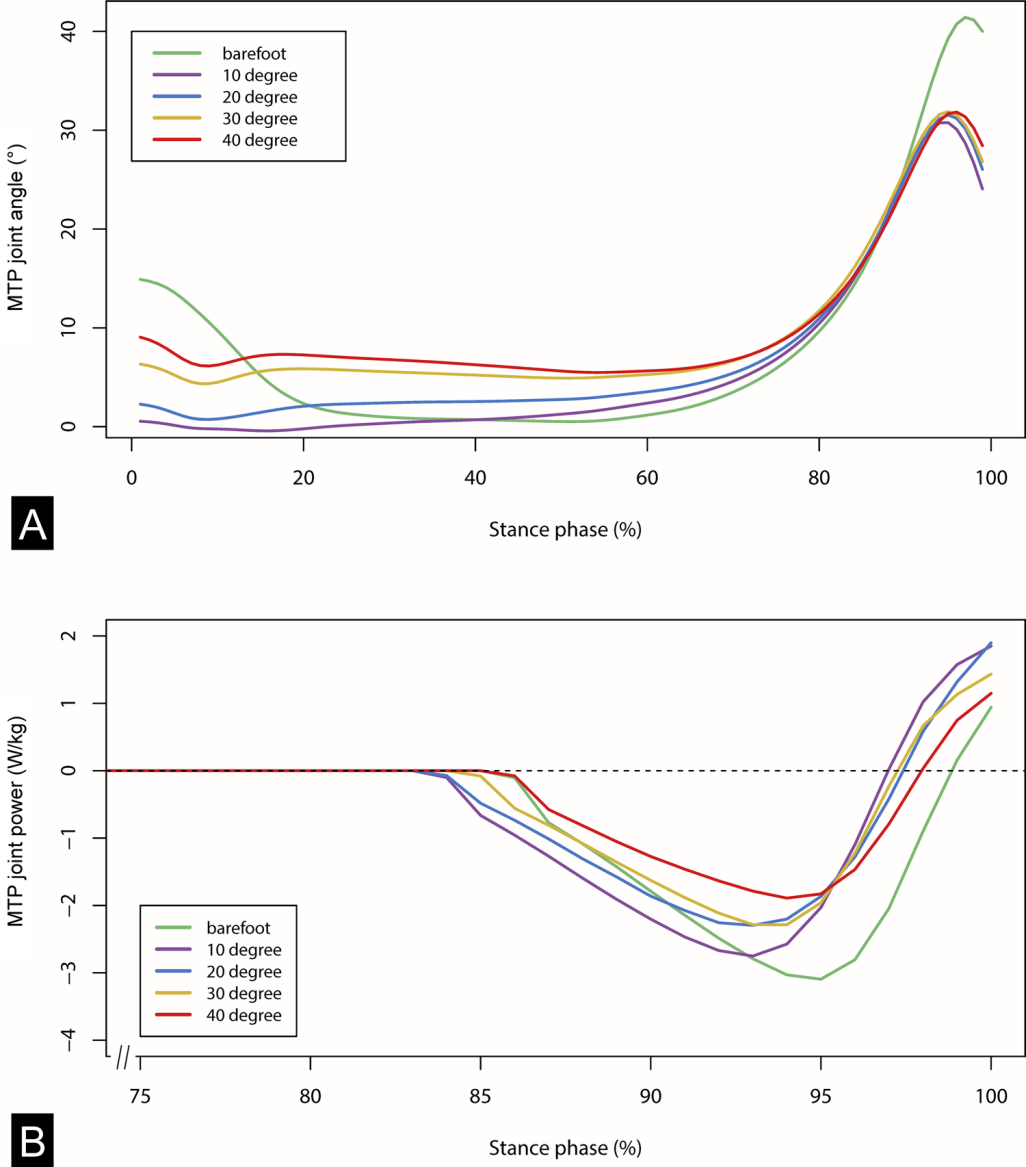
(**A**) Mean temporal profiles of the metatarsophalangeal (MTP) joint dorsiflexion angle during normalized stance phase duration. Subjects walked barefoot (green) and in four curved sandal conditions (purple: 10°, blue: 20°, yellow: 30°, red: 40°). Toe springs increased MTP joint dorsiflexion at midstance but decreased dorsiflexion at the end of stance. (**B**) Mean temporal profiles of the MTP joint power during normalized stance phase duration.

Quasi-stiffness of the midtarsal joint during midstance (defined as the slope of the joint’s moment–angle relationship) was computed using the MLA joint angles and corresponding joint moment^43^. The quasi-stiffness was computed only when the COP was anterior to the midtarsal joint center (defined by the marker on the sustentaculum tali) until the heel left the ground. MTP joint moment and power were defined to be zero until the resultant GRF vector moved anterior to the MTP joint center^19,35^. Power was calculated using the following equation: $P = M\times \omega$, where M is the moment and ω is the angular velocity at the MTP joint, derived from the kinematic data (Fig. 3B). Negative and positive work were then quantified by taking the integral of power over time of push-off (i.e., from the moment when the resultant GRF vector originates anterior to the MTP joint center to the moment the toes lifted off the ground). Distance of travel of COP after it moved anterior to the MTP joint was also quantified. All calculations were performed using Visual3D, and custom MATLAB (The MathWorks, Natick, MA, USA) and R (R Core Team 2019, Vienna, Austria) scripts.

### Statistical analysis

Means and standard deviations (mean ± SDs) were calculated and a Shapiro–Wilk test of normality was performed for all variables. Further comparison of kinematic and kinetic measurements between the barefoot and the four sandal conditions was performed using a one-way repeated measures ANOVA for normally distributed outcome parameters, or a Friedman test for those measurements that were not normally distributed. When a significant main effect between conditions was observed, Bonferroni-adjusted post-hoc analysis was performed. For the non-normally distributed measurements, a Wilcoxon signed-rank test was performed. For all tests significance was set at α = 0.05, using IBM SPSS Statistics, version 25 (IBM, Armonk, New York, USA).

## Results

At midstance, the increasingly angled toe springs kept the toes at slightly but significantly increasing degrees of dorsiflexion (p < 0.05, repeated measures ANOVA). Bonferroni-adjusted post-hoc analysis revealed significant differences in MTP joint dorsiflexion between the different toe spring conditions (Table 1). However, the total degree of toe dorsiflexion at midstance was relatively small, reaching a maximum of 6.28° (± 2.35°) with the 40° sandal (Fig. 3). Variations in toe spring angle did not have a significant effect on midtarsal joint quasi-stiffness (p > 0.05, Friedman ANOVA).

**Table 1:** Mean values and standard deviations of medial longitudinal arch (MLA) and metatarsophalangeal (MTP) joint kinematics and kinetics during stance phase for walking barefoot and in sandals with varying toe spring angles.

	Barefoot	10° sandal	20° sandal	30° sandal	40° sandal	Main effect p-Value
MTP joint dorsiflexion at midstance (°)	0.49 (0.72)	1.23 (1.74)	3.01 (1.91) ^bf,10^	5.41 (2.28) ^bf,10,20^	6.28 (2.35) ^bf,10,20,30^	> 0.001
Time of stance phase when COP aligns with MTP joint (°)	82.31 (7.11)	79.77 (6.19)^bf^	80.54 (7.31)	81.38 (6.70)^10^	82.76 (7.22)^10^	0.021
MTP joint dorsiflexion at moment when COP aligns with MTP joint (°)	14.42 (9.87)	11.88 (8.17)^bf^	13.09 (7.96) ^10^	14.60 (9.09) ^10,20^	15.82 (8.93) ^10,20,30^	0.006
Peak MTP joint dorsiflexion (°)	41.43 (5.14)	30.88 (5.82) ^bf^	31.54 (5.33) ^bf^	31.89 (5.93) ^bf^	31.83 (5.93) ^bf^	> 0.001
Total MTP joint range (°)	27.94 (10.23)	19.72 (7.71) ^bf^	18.93 (7.58) ^bf^	17.94 (7.93) ^bf,10^	16.58 (8.68) ^bf,10,20^	> 0.001
Peak MTP joint velocity (rad/s)	5.44 (1.95)	3.36 (1.40)^bf^	3.44 (1.33)^bf^	3.37 (1.01)^bf^	2.91 (1.98)^bf^	> 0.001
Peak MTP joint moment (Nm)	7.16 (3.67)	10.15 (4.54)^bf^	8.88 (4.47)	8.56 (4.11)	7.54 (3.92) ^10^	0.003
Distance of travel of COP distal to MTP joint (mm)	30.72 (8.16)	39.26 (18.31)^bf^	38.26 (17.3)	34.20 (10.89)	30.55 (11.16)^10,20,30^	0.022
Negative work (J)	− 2.81 (2.08)	− 2.74 (2.12)	− 2.38 (2.26)	− 2.13 (1.79)	− 1.81 (1.65) ^bf,10^	0.011
Positive Work (J)	0.06 (0.10)	0.28 (0.33)^bf^	0.22 (0.25)^bf^	0.16 (0.20)	0.11 (0.09)	0.016
Midtarsal joint quasi-stiffness at midstance (Nm/°)	0.20 (0.04)	0.22 (0.07)	0.20 (0.05)	0.22 (0.06)	0.22 (0.05)	0.262

^bf^Value significantly different from barefoot.

^10^Value significantly different from 10° toe spring angle.

^20^Value significantly different from 20° toe spring angle.

^30^Value significantly different from 30° toe spring angle. All values are significant at the p < 0.05 level.

At push-off, a notable difference of 10.55° for peak MTP joint angle was found between the barefoot and 10° sandal condition. Contrary to expectation, no differences were found for peak MTP joint angle between all sandal conditions (p > 0.05, repeated measures ANOVA) (Fig. 3, Table 1). However, total range of MTP joint dorsiflexion angle changed between the barefoot and all sandal conditions. Between barefoot and 10° sandal condition, the total MTP joint range dropped significantly by 29.42% (p < 0.05, Friedman ANOVA). With increasing toe-spring angle, the total MTP joint range further decreased by up to 15.92% between the 10° (19.72° ± 7.71°) and 40° sandals (16.58° ± 8.68°) (p < 0.05, Friedman ANOVA). The change in total MTP joint range corresponds well with the MTP joint dorsiflexion angle at the moment when the COP passed the MTP joint center. The MTP joint angle at that moment increased with increasing toe spring angles, from 11.88° ± 8.17° in the 10° sandal to 15.82 ± 8.93° in the 40° sandal (p < 0.05, Friedman ANOVA). Along with the changes in total range of MTP joint dorsiflexion angle, significant differences were found in the time when the COP passed the MTP joint center during stance phase (p < 0.05, Friedman ANOVA). The COP passed the MTP joint center significantly earlier in the 10° sandal (79.77 ± 6.19% stance phase) compared to barefoot (82.31 ± 7.11% stance phase) and the 40° sandal (82.76 ± 7.22% stance phase).

**Figure 4:**
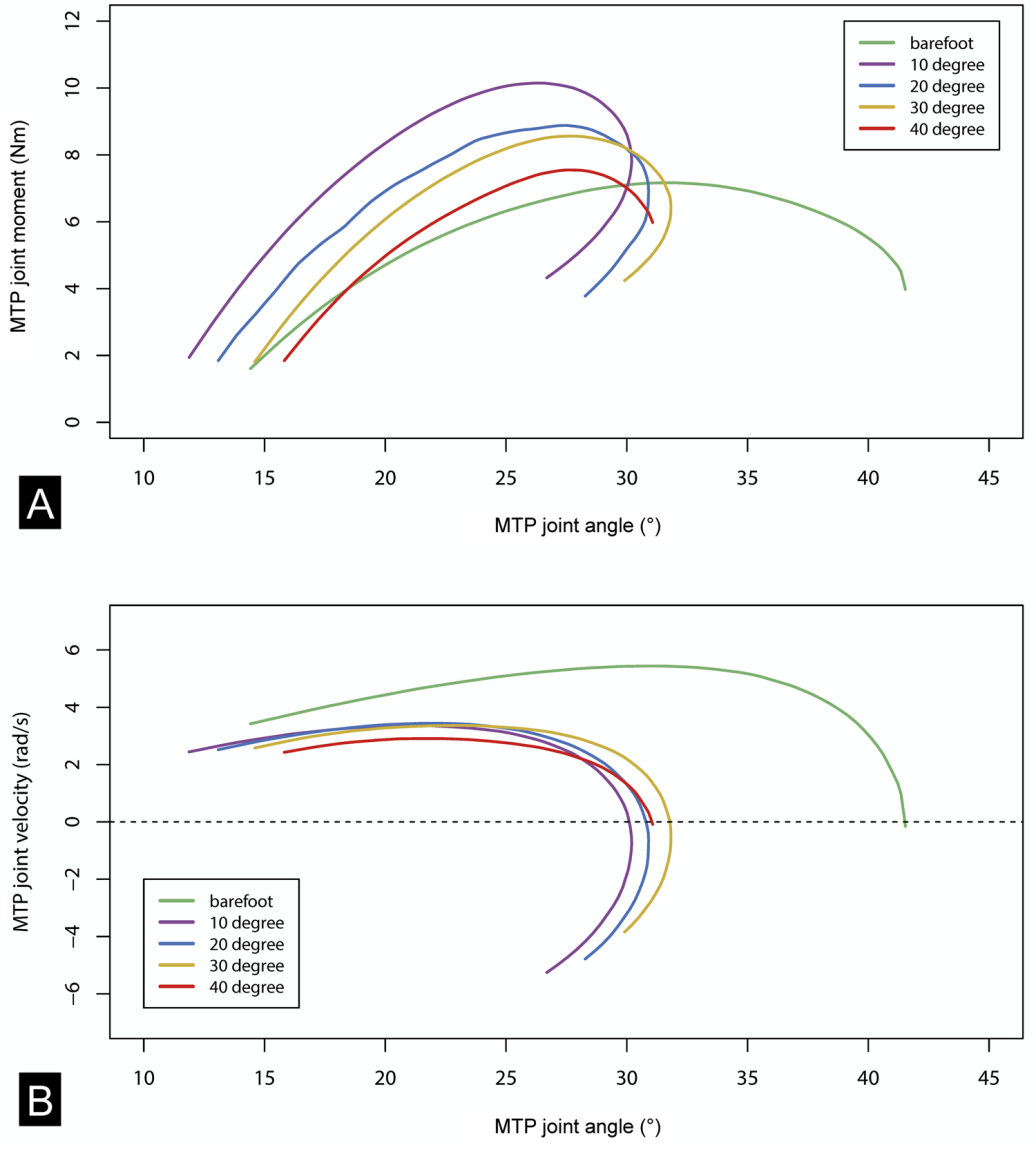
Toe springs did not affect the metatarsophalangeal (MTP) joint velocity but did change the acting MTP joint moment during propulsive phase. (**A**) plots the MTP joint angle against the MTP joint moment. (**B**) plots the MTP joint angle against the MTP joint velocity. Both plots display the mean values of all participants during walking barefoot (green) and in four curved sandal conditions (purple: 10°, blue: 20°, yellow: 30°, red: 40°).

While peak MTP joint velocity was significantly higher in the barefoot condition, there were also no differences in peak angular velocity between the sandal condition (p > 0.05, Friedman ANOVA) (Fig. 4A, Table 1). The most interesting finding to emerge from the data during propulsive phase is that peak moment increased with the 10° sandals compared to the barefoot condition, but decreased gradually with increasing toe spring angle by up to 31.63% between the 10° and 40° sandals (Fig. 4B, Table 1). This behavior in peak moment is likely related to the fact that the distance of travel of COP distal to the MTP joint increased between barefoot and 10° sandal condition, but decreased significantly with increasing toe-spring angle by up to 22.26% between the 10° (39.26 ± 18.31 mm) and 40° sandals (30.55 ± 11.16 mm)(p < 0.05, Friedman ANOVA) (Table 1).

Negative MTP joint work was greatest in the barefoot and 10° sandal (− 2.81 ± 2.08 and − 2.76 ± 2.12 J, respectively) and least in the 40° sandal (− 1.81 ± 1.65 J). A comparison between the sandals showed a gradual decrease by 2.5%, 15.3%, 24.2%, and 35.6% relative to the 10° sandal for the 20°, 30° and 40° sandals, respectively (p < 0.05, Friedman ANOVA) (Table 1). Bonferroni-adjusted post-hoc analysis revealed a significant difference between the 10° and 40° sandals. Positive MTP joint work was significantly different between barefoot and 10° as well as 20° sandals (p < 0.05, Friedman ANOVA). No significant difference was found between the sandal conditions, but the data indicate a gradual, slight decrease from the 10° sandal (0.28 ± 0.33 J) to the 40° sandal (0.11 ± 0.09 J) (Table 1).

## Discussion

The present study was designed to model and then test the effects of toe springs in shoes on foot biomechanics during walking. We hypothesized that toe springs would increase the stiffness of the medial longitudinal arch during midstance by engaging the windlass mechanism. We further hypothesized that the negative work at the MTP joints would decrease during the propulsive phase because toe springs would reduce the total angle through which the toes rotate. While we did not find any change in arch stiffness due to toe springs, our results indicate that toe springs decrease the negative work at the MTP joints during push-off. As expected, greater toe spring angles decreased the total range of MTP joint rotation. Linked to this, we found that more angled toe springs delayed the time when the COP passed the MTP joint center and reduced the distance traveled by the COP anterior to the MTP joint. As a result of these kinematic changes, the corresponding joint moment and resulting negative work performed decreased. Comparison of results from the toe spring conditions to those of the barefoot condition revealed that toe springs seem to be able to compensate for the negative effects of stiff shoes on MTP work requirements.

The windlass mechanism predicts that dorsiflexion of the toes increases tension on the plantar aponeurosis, which stiffens the longitudinal arch as a whole^12^. However, the small increases in MTP joint dorsiflexion angles caused by toe springs in this study did not affect measured arch stiffness. This result corresponds to the findings of Welte et al.^14^, who used static loading to compress the arches of sitting participants at different toe dorsiflexion angles, and found that engaging the windlass mechanism did not increase arch stiffness. A possible explanation might be the flexibility of the plantar aponeurosis, which stretches as the arch is loaded^44–46^. Thus, our results can be interpreted as further evidence that the windlass mechanism does not contribute substantially to stiffening the longitudinal arch at midstance. Another possible explanation for the lack of toe spring effect on arch stiffness at midstance in our study could have been the relatively small MTP joint dorsiflexion angles achieved in the toe spring conditions. Although we designed sandals with toe spring angles of up to 40°, and a bending stiffness that is similar to conventional shoes, the maximum MTP joint dorsiflexion angle caused by toe springs was less than 10°. Although we do not know why these angles were so low, it is possible that during midstance, when the arch is compressed by force from above and the plantar aponeurosis tenses, the windlass is unwound. This unwinding of the windlass, described as a ‘reverse windlass’^12,15^, could plantarflex the toes at the MTP joint and counteract the curvature of the toe spring. However, this mechanism needs further testing.

In contrast to what we document at midstance, our results indicate that during the propulsive phase toe springs affect MTP joint dynamics, as evidenced by the significant decrease in negative work associated with increasing toe spring angles. Paradoxically, negative work was highest during walking barefoot and with 10° sandals. However, these values were highest in these conditions for different reasons. When barefoot, participants achieved high peak MTP dorsiflexion angles, necessitating high angular velocity and hence high magnitudes of negative work. In contrast, the 10° sandal showed a reduced MTP dorsiflexion angle but caused the COP to move more distally during toe-off, effectively increasing the acting moment and hence negative work. The distal shift of the COP in sandals is likely a consequence of pushing off against a relatively stiff platform in the sole, which is also reflected by an earlier passing of the COP relative to the MTP joint center. These findings are broadly consistent with other studies linking differences in MTP joint dynamics with shoe stiffness^47–50^. While there were no differences in peak MTP dorsiflexion angles across sandals with different degrees of toe spring, there were significant differences in time when the COP passed the MTP joint center. With increasing toe spring angles, the COP passed the MTP joint center later in stance phase. The delay in timing might explain the reduced total MTP joint range and magnitude of distal COP motion. Along with these findings, our results show a significant decline in negative work coincident with the reductions in MTP joint range and COP travel due to increasing toe spring angles. In addition, the data suggest a gradual downturn of the peak angular velocity with increasing toe spring angles, but this difference is not significant between conditions. Thus, toe springs seem to counteract the negative effects of stiff shoes on MTP work requirements. While stiff shoes do stiffen the MTP joints, toe springs might compensate for the effects of increased COP travel distal to the MTP joints, and further reduce total MTP joint range and possibly peak angular velocity, thereby reducing negative work.

The decrease in negative work at the MTP joints suggests that intrinsic foot muscles have to perform less eccentric muscle work to control MTP joint dorsiflexion during the propulsion phase of gait^19^. Farris et al.^15,19^ found that the intrinsic foot muscles play an important role in helping to stiffen the MTP joints as they are being dorsiflexed at the end of a step in walking and running. By reducing moments at the MTP joints, toe springs likely relieve the intrinsic foot muscles of some of the work necessary to stiffen these joints. While the differences in joint work among conditions measured in this study are relatively small, the intrinsic foot muscles themselves are also small, meaning that a higher proportion of the available fascicles will need to be contracted to produce a given amount of energy than in larger limb muscles. Furthermore, these small differences in muscle work likely add up to substantial differences over time when considering that the average individual in industrialized countries takes 4,000 to 6,000 steps per day^51^. Thus, habitually wearing shoes with toe springs could inhibit or de-condition the force generating capacity of intrinsic foot muscles. While the direct consequences of weak foot muscles are not fully known, it is likely that they could increase susceptibility to flat foot and associated problems such as plantar fasciitis^21,23^. This painful condition, which is recognized as an injury caused by excessive and repetitive loading of the foot’s longitudinal arch^26^, is the most commonly treated foot problem among habitually shod populations^52^. Farris et al. ^15^ recently suggested that the intrinsic foot muscles contract to prevent strain in the plantar aponeurosis under high loads, and thus weakening the intrinsic foot muscles may limit their ability to perform this function.

It is crucial to bear in mind that the possible link between toe springs and plantar fasciitis needs further testing. Unfortunately, this study included only habitual shoe users, whose feet might already have been adapted to shoes with toe springs. Further studies are needed to investigate the effect of toe springs on habitually barefoot individuals. Additional limitations that need further testing are walking speed and gaits. While this study tested only one walking speed, and did not look at running, future investigations should also test greater speeds that increase the demands on arch stability and muscle activity. This study also did not assess intrinsic foot muscle activity; therefore, uncertainty remains regarding if changes in muscle activity reflect the alterations in MTP joint work. It is possible that the intrinsic foot muscles contract isometrically as the toes are being dorsiflexed during walking, and that changes in power at the MTP joints among toe spring conditions reflect differences in elastic energy storage and release, rather than changes in intrinsic foot muscle work. Recent static loading experiments from Kelly et al.^53^ have suggested that intrinsic foot muscle fascicles actually contract concentrically at high loads, but further research is necessary to determine whether this holds true during walking and running. Additional caution should be taken when interpreting the MTP joint kinetics. MTP moment, power and work were calculated from the moment when the COP passed anterior to the MTP joint. This approach has been used in previous studies (Rolian et al., 2009; Farris et al., 2019), but might slightly overestimate the moment and power calculations at the toe joints when compared to more complex methods requiring independent force measurements from multiple forceplates^54^. Nevertheless, we expect this effect would be consistent across conditions used in the present study, and therefore should not affect our overall conclusions.

Notwithstanding these limitations, we conclude that toe springs have important heretofore unrecognized biomechanical effects on foot function that merit consideration, especially since they have become increasingly exaggerated in modern athletic shoes^55^. As shown here, toe springs can alter the natural biomechanics of the foot during walking principally by altering total work at the MTP joint and thus potentially reducing the work required by the intrinsic foot muscles. Consequently, while toe springs may increase comfort by reducing the effort of the foot muscles, they may increase susceptibility to plantar fasciitis and other foot-related problems. That said, considerably more work will need to be done to understand more fully the effects of toe springs on foot function and overall gait. Future studies might explore the impact of toe springs in combination with other shoe features, including insoles, shoe stiffness, and cushioning. Also, future research should investigate how toe springs could affect more general aspects of gait such as center of mass mechanics, which have previously been shown to be affected by MTP joint stiffness and shoe sole curvature^2,3^. Studies could also explore additional walking speeds and running to provide a more comprehensive understanding of how toe springs affect gait that might help improve footwear design and use. Finally, future research should incorporate techniques such as EMG to explore how the mechanical effects of toe springs observed here relate to actual neuromuscular output and control during gait.
